# Evaluating the Adaptation Process of Sandfly Fauna to Anthropized Environments in a Leishmaniasis Transmission Area in the Brazilian Amazon

**DOI:** 10.1093/jme/tjw182

**Published:** 2016-12-08

**Authors:** Ingrid N. G. Rosário, Andrey J. de Andrade, Raphael Ligeiro, Ricardo Ishak, Ivoneide M. Silva

**Affiliations:** 1 Laboratório de Parasitologia, ICB, Universidade Federal do Pará, Belém, Pará, Brasil (ingridgarciiar@gmail.com; ivisilva8@yahoo.com.br); 2 Laboratório de Parasitologia Molecular, Departamento de Patologia Básica, Setor de Ciências Biológicas, Universidade Federal do Paraná, PR, Brasil (andreyandrade@ufpr.br); 3 Laboratório de Ecologia e Conservação, ICB, Universidade Federal do Pará, Belém, Pará, Brasil (ligeirobio@gmail.com); 4 Laboratório de Virologia, ICB, Universidade Federal do Pará, Belém, Pará, Brasil (rishak@ufpa.br),

**Keywords:** phlebotominae, leishmaniasis, vector environmental adaptation, Brazilian Amazon

## Abstract

Phlebotomines (Diptera: Psychodidae) are vectors of several etiological agents of human and animal diseases, including protozoans of the gender *Leishmania*. Precarious socioeconomic conditions and uncontrolled population growth directly influence the transmission risk of parasites and the urbanization of vector species, previously restricted to wild environments. The Marajó Archipelago is considered a high incidence area of leishmaniasis in the Brazilian Amazon. However, it is poorly studied. The aim of this study was to assess the adaptation processes of phlebotomine species to anthropized environments in this region. For this purpose, the phlebotomine fauna was compared between three municipalities of the Marajó Archipelago: Anajás, Portel, and São Sebastião da Boa Vista. To survey the phlebotomine fauna, CDC (Center for Disease Control) light traps were installed in the wild areas and in the intra and peridomiciliary areas of rural and urban environments. The environments studied presented a diversified phlebotomine fauna, with higher richness in the wild environment (15 species), followed by the rural (seven species), and finally, the urban environment (three species). A migration of wild fauna to the adjacent anthropized areas (rural environment) and to urban areas was observed, evidencing the adaptation process of this vector to anthropized environments in the studied region. Thus, our study evidenced that the disorganized human occupation and utilization of the landscape might cause the invasion of urban areas by wild populations of phlebotomines, in this way enabling the settlement of urban leishmaniasis transmission cycles.

Phlebotomines (Diptera: Psychodidae: Phlebotominae) are vectors of several etiological agents of human and animal diseases, such as protozoans of the genera *Leishmania* Ross, 1903 (Ward 1977) and *Trypanosoma* Gruby, 1843 (Arias and Freitas 1982), bacteria of the gender *Bartonella* Barton, 1909 (Schultz 1968), and some arboviruses (Tesh et al. 1974).

*Leishmania* (Protozoa: Kinetoplastida: Trypanosomatidade) cause a group of infectious diseases called Leishmaniases (Dias Júnior 2008), and >20 infectious species for humans and animals (WHO 2014) have been reported. These diseases attack mostly people from Africa, Asia, and Latin America, and they are considered endemic in >98 countries. An estimated 0.2–0.4 million new cases of visceral leishmaniasis (VL) and 0.7–1.2 million new cases of New World cutaneous leishmaniasis (NWCL) have been reported worldwide per year (WHO 2014).

New World cutaneous leishmaniasis is reported in all states of Brazil, and around 159,300 new cases were reported in the period ranging from 2007 to 2013. Brazilian northern region accounted for 41% (65,272) of the cases, indicating that this is an expanding zoonosis (Ministry of Health 2014). The VL cases have also increased, mainly in the northern, southern, and mid-western regions (Oliveira et al. 2011). In Pará State, 25,642 NWCL cases and 2,224 VL cases were confirmed between 2007 and 2013 (Ministry of Health 2014). The occurrence of VL and NWCL necessarily follows the distribution of vector insects (Carvalho et al. 2010). Currently are described 989 phlebotomine species in the world, of which 531 occur in the Americas. Out of those, 276 have been recorded in Brazil, representing 28% of the world's total and 52% of the species that occur in the Americas (Shimabukuro and Galati 2011, Almeida et al. 2015). Approximately 20 of those species are incriminated in the transmission of *Leishmania* species to humans and animals (Ready 2013).

Leishmaniases used to be considered strictly wild vector-borne diseases. However, over the past few years, they have proven to be important issues of urban public health in several countries. This occurs not only because of the emergence of diseases in new areas, but also due to its reappearance in old outbreak areas (Barata et al. 2005). Some of the factors that have caused these zoonoses to suffer an urbanization process are environmental changes and the constant migration movement of populations from the outskirts of the cities to large centers (Gállego 2014). Therefore, the expansion of these endemics is strongly related to environmental issues, such as land occupation and deforestation, and this has been aggravated by the population's socioeconomic conditions; the main income sources in impoverished communities are vegetable extraction, fishery, hunting, and other activities that expose people to vectors (Novo 2011).

One of the anthropic activities that are closely related to the increased incidence of tropical diseases, especially leishmaniasis, is the deforestation of forest areas for commercial purposes, such as cattle breeding, agriculture, timber extraction, and even road and housing construction, typically with precarious infrastructure. In any of these situations, the physical environment is changed due to forest fragmentation, with consequent impacts on the wild fauna, which are blood feeding sources for phlebotomines. Thus, anthropic changes on the environment make these insects search for new blood feeding sources, such as dogs and humans (Patz et al. 2004).

Due to the anthropization of landscapes, some phlebotomine species, which used to have a wild behavior, are adapting to changes caused by humans and have been found near human dwellings and in plantations (Marzochi et al. 1985). This has led to the emergence of new epidemiological patterns, such as peridomiciliary transmission (Forattini 1973). Some vector species are able to adapt to the new conditions generated by environmental degradation, as is the case of *Nyssomyia whitmani* (Antunes & Coutinho, 1939), which might be found in great abundance in anthropized areas (Costa et al. 2007).

Studies on phlebotomine fauna are important to determine epidemiological potential and to elaborate regional control strategies for the populations of these insects. In this context, this study assessed the adaptation process of phlebotomine species to anthropized environments in an area of the Brazilian Amazon with a high incidence of leishmaniasis (Marajó Archipelago), comparing richness, abundance, and species composition (assemblages) of phlebotomines between preserved forest, rural, and urban environments.

## Materials and Methods

### Study Area

**Fig. 1:**
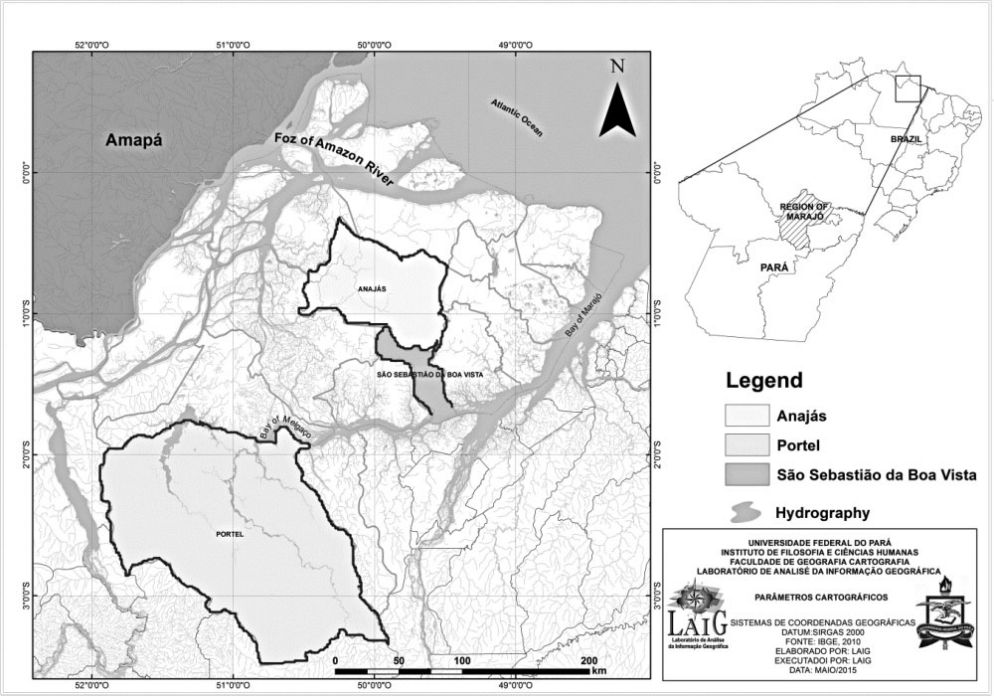
Map of the Marajó Archipelago, located in Pará State, Brazil, highlighting the municipalities studied: Anajás, Portel, and São Sebastião da Boa Vista.

The Marajó Archipelago, situated in the northernmost part of Pará State, is composed of a group of islands that form the largest marine–fluvial island in the world (49,606 km^2^), of which Marajó island is the best known. Its limits are: the discharge of the Amazon River and the Atlantic Ocean to the north, Marajó Bay to the east, the Amazon River to the south, and the discharge of the Amazon River to the west (Lima et al. 2005; Fig. 1).

Marajó Island has ∼350 thousand inhabitants in 13 municipalities, constituting one of the poorest regions of Pará State (Plano de Desenvolvimento Sustentável do Arquipélago de Marajó [PDTSAM] 2007). The economic structure of the region is based on cattle breeding, artisanal fishery, and woodcutting. Population density is low but its growth rate has been increasing.

Local human populations have many socioeconomic problems, such as poverty, high illiteracy rate, no basic sanitation, and precarious medical service. Among the diseases present in this island, the highlights are leishmaniasis and malaria. The epidemiological situation of leishmaniasis in the municipalities of the Marajó Archipelago is quite concerning, and there has been an increase in the number of cases, mainly of NWCL (Ministry of Health 2014).

To study the phlebotomine fauna of the Marajó Island, three municipalities with a history of leishmaniasis were chosen: Anajás (00° 59′12″ S e, 49° 56′24″ W), São Sebastião da Boa Vista (01° 43′03″ S e, 49° 32′27″ W), and Portel (01° 56′08″ S e, 50° 46′16″ W; Fig. 1).

### Sample Design

Phlebotomine samplings were carried out for 4 mo in 2012 (September to December), using CDC (Center for Disease Control) light traps (Sudia and Chamberlain 1962). The traps were placed 1.5 m from the ground, and remained active for ∼12 h (6:00 p.m.–6:00 a.m.).

To assess the dispersal of phlebotomines to anthropic areas, three transects were defined in each municipality, each containing a wild, a rural, and an urban environment, therefore representing a gradient of anthropic disturbances. The three types of environments were characterized as follows:

- Wild—Areas with dense vegetation, with sparse residences or lack thereof. In all municipalities, wild environments were partially preserved areas, with large-sized trees cut through by streams and with low light. Small anthropic changes were evident in some parts, such as trail openings and house construction.
- Rural—Intermediate areas between wild and urban environments, with sparse houses, most of them made of wood. The main activity of the rural population is chicken and pig farming, and there are many domestic animals, mainly dogs, and also wild animals, mainly rodents and marsupials.
- Urban—Areas with a higher concentration of residences, paved streets, and artificial lighting. The urban areas in the three municipalities are characterized by little vegetation and the presence of brick houses with electric power. There were many domestic animals moving around on the streets and chickens in the backyards of some residences.

Samplings were carried out in wild environments both in the border and in the inner part of the forests. In rural and urban environments, samplings were carried out both in the peridomiciliary areas (backyards, chicken coops, pigsties etc.) and intradomiciliary areas (dormitories and kitchen). In each transect, traps were installed in the wild environment (three) and in the intra (three) and peridomiciliary areas (three) of rural and urban environments, totaling 15 traps per transect and 45 traps per municipality (each trap corresponded to 12 h of sampling). The traps were installed within a minimum distance of 30 m from each other to ensure independence between samples. Data obtained in each set of three traps were grouped to comprise one sample of each environment type per transect, and this was our sampling unit.

Percentage of vegetation cover at each sampling unit was measured through Landsat imagery. The Landsat imagery have undergone a process of atmospheric correction using ATCOR algorithm in PCI Geomatica 9.1 program. Next, it was calculated the Red Edge Normalized Difference Vegetation Index (NDVI Red Edge) using the bands spectrum of near infrared and red from RapidiEye images in ArcGIS software, version 10.1 and Envi 5.2.

**Fig. 2:**
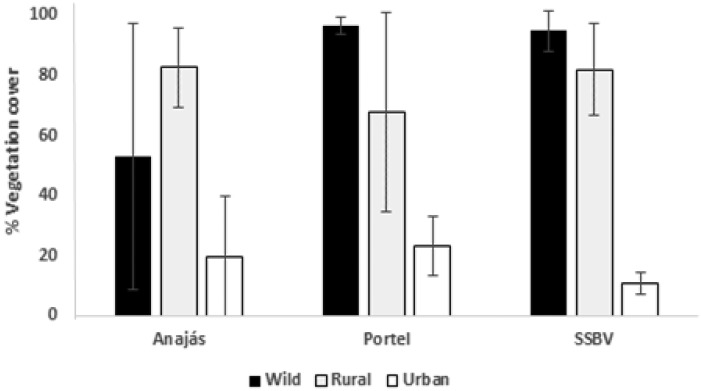
Mean percentage of vegetation cover (± SD) observed at each environment and municipality sampled in the Marajó Archipelago, Pará, Brazil.

Results are presented in Fig. 2. Wild and rural areas did not differed, and both were statistically higher than urban areas (ANOVA, *F*_ _= 22.593; df = 2, 18; *P* < 0.001). Municipalities did not differ (ANOVA, *F*_ _= 0.755; df = 2, 18; *P* = 0.485), neither the interaction between environmental types and municipalities was significant (ANOVA, *F*_ _= 1.708; df = 4, 18; *P* = 0.192).

Insects collected were stored in properly identified tubes for later quantification and identification according to the classification proposed by Galati (2003).

### Statistical Analysis

Taxonomic richness and abundance of individuals were compared between the types of environment and the sampled municipalities using a factor analysis of variance (two-way ANOVA) followed by post hoc Tukey's HSD tests for pairwise comparisons. Significance values were assessed under α = 0.05. Gross values of both response variables were log transformed (log_10_ X + 1) to diminish discrepancies in the data and increase homogeneity in residue variance.

Permutational multivariate analysis of variance (PERMANOVA) was employed in a factorial design to compare the assemblages of phlebotomines between sampled environments and municipalities, followed by pairwise comparisons between treatments. Sampling sites were compared using the Euclidean distance, and abundance values were previously log transformed (log_e_ X + 1). 10,000 permutations were performed to access the significance of the observed distances (α = 0.05). An indicator species analysis (Dufrene and Legendre 1997) was performed to identify indicator species in the three environments, i.e., species whose relative frequencies and abundances were particularly high in one type of environment. 10,000 permutations were performed to access the significance of the indication values (IndVal) observed for each species, with α = 0.05. Tests were performed using the R Program.

## Results

### Comparing Assemblages Between Municipalities

A total of 860 phlebotomines were collected between males (511 specimens; 59.42% of the total number of individuals) and females (349 specimens; 40.58%) in the three municipalities studied. Eighteen species were identified, grouped in 10 genera: *Bichromomyia*, *Brumptomyia*, *Evandromyia*, *Micropygomyia*, *Nyssomyia*, *Pintomyia*, *Psathyromyia*, *Sciopemyia*, *Thricopygomyia*, and *Trichophoromyia.* The genera, *Evandromyia* showed the highest taxonomic richness, with five species. The species *Ev. walkeri* was predominant, representing 69.07% of the total collected, accounting for 76.6% of the individuals collected in Portel, 70% of individuals collected in São Sebastião da Boa Vista (SSBV), and 45% of the individuals collected in Anajás. This species was followed by *Ev. infraspinosa* (15.17%) and *Ny. antunesi* (4.78%) in the total number of individuals collected (Table 1).

**Table 1:** Species of phlebotomines captured in three municipalities of Marajó Island (Pará, Brazil), by municipality and sampling environment (wild, rural, and urban)

Species	Anajás			Portel			SSBV				
	Wild	Rural	Urban	Wild	Rural	Urban	Wild	Rural	Urban	Total	(%)
*Evandromyia walkeri*	44	40	1	156	296	1	51	1	2	592	68.84
*Evandromyia infraspinosa*	44	0	3	82	1	0	0	0	0	130	15.12
*Nyssomyia antunesi^a^*	11	3	3	20	3	0	1	0	0	41	4.77
*Micropygomyia rorotaensis*	20	1	0	4	0	0	2	0	0	27	3.14
*Sciopemyia sordellii*	7	1	0	13	2	0	2	0	0	25	2.91
*Bichromomyia flaviscutellata^a^*	0	0	0	4	0	0	16	0	0	20	2.32
*Nyssomyia yuilli yuilli*	4	0	0	0	0	0	0	0	0	4	0.46
*Psathyromyia aragaoi*	2	0	0	2	0	0	0	0	0	4	0.46
*Psathyromyia dendrophila*	4	0	0	0	0	0	0	0	0	4	0.46
*Brumptomyia* sp.	1	0	0	1	0	0	0	0	0	2	0.23
*Micropygomyia pilosa*	2	0	0	0	0	0	0	0	0	2	0.23
*Pintomyia nevesi*	0	0	0	2	0	0	0	0	0	2	0.23
*Thricopygomyia trichopyga*	0	0	0	2	0	0	0	0	0	2	0.23
*Evandromyia bacula*	0	0	0	1	0	0	0	0	0	1	0.12
*Evandromyia furcata*	0	0	0	0	0	0	0	1	0	1	0.12
*Evandromyia monstruosa*	0	0	0	1	0	0	0	0	0	1	0.12
*Psathyromyia* (series *shannoni*) sp.	0	0	0	0	0	0	1	0	0	1	0.12
*Trichophoromyia* sp. n.	0	1	0	0	0	0	0	0	0	1	0.12
Subtotal	139	46	7	288	302	1	73	2	2		
Total	192			591			77			860	100

^*a*^Species with recognized importance in the transmission of infectious agents.

The number of males captured was higher than the number of females in the municipalities of Anajás (106M/86F) and Portel (372M/219F), while in SSBV, a higher number of females was observed (32M/45F).

Comparing the phlebotomine fauna in the three municipalities studied, we observed that the interaction between municipalities (Portel, Anajás, and SSBV) and types of environments (wild, rural, and urban) was not significant to explain taxonomic richness (ANOVA, *F*_ _= 1.81; df = 4, 18; *P* = 0.17), abundance of individuals (ANOVA, *F*_ _= 0.41; df = 4, 18; *P* = 0.79), or phlebotomine assemblage composition (PERMANOVA, Pseudo-*F*_ _= 1.01; df = 4, 18; *P* = 0.44). Therefore, the effects of each factor could be interpreted separately.

**Fig. 3:**
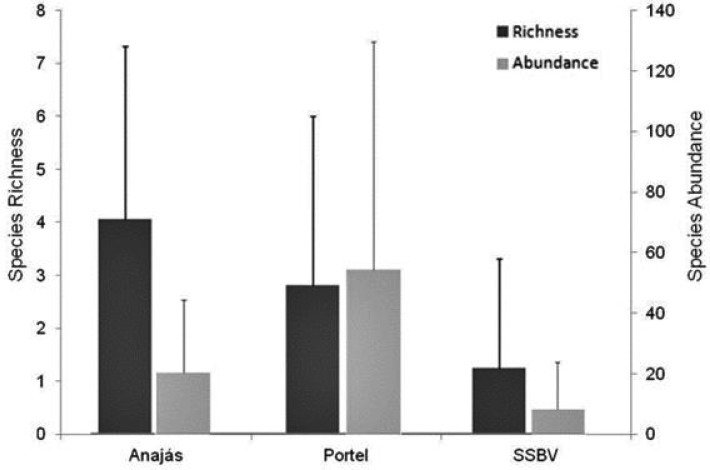
Mean species richness and abundance of individuals (± SD) observed in the three municipalities studied in the Marajó Archipelago, Pará, Brazil.

Portel showed the highest abundance and richness of phlebotomines collected (591 individuals, distributed in 12 species), followed by Anajás (192 individuals, distributed in 10 species) and SSBV (77 individuals, distributed in seven species). However, no significant difference in taxonomic richness (ANOVA, *F* = 1.89; df = 2, 24; *P* = 0.17) and in mean abundance of individuals (ANOVA, *F*_ _= 1.64; *P* = 0.21) was observed between the municipalities (Fig. 3).

**Fig. 4:**
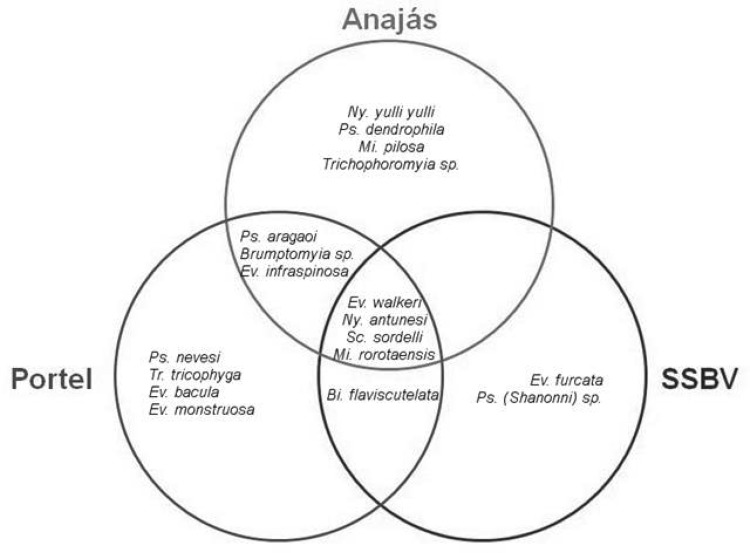
Distribution of the collected species, by municipality studied.

Phlebotomine species composition was very similar between the municipalities, and it didn't show significant difference (PERMANOVA, Pseudo-*F* = 1.36; df = 2, 18; *P* = 0.23). Of the 18 collected species, four occurred in the three municipalities, three were common to Portal and Anajás, and one was common to Portel and SSBV (Fig. 4). Only two species occurred exclusively in SSBV, while the municipalities of Portel and Anajás had four exclusive species each (Table 1, Fig. 4).

### Comparing Assemblages Between Environments

In all municipalities, the presence of phlebotomines was observed in the three environments assessed. In SSBV, 94.8% of the individuals were collected in the wild environment. However, a small number of individuals were also collected in the rural (2.6%) and urban (2.6%) environments. In Portel, individuals collected were concentrated in the wild and rural environments (48.7% and 51.1% of individuals, respectively), and only one individual was collected in the urban environment. In Anajás, 72.0% of individuals were captured in the wild environment, 24.3% in the rural environment, and 3.7% in the urban environment.

**Fig. 5:**
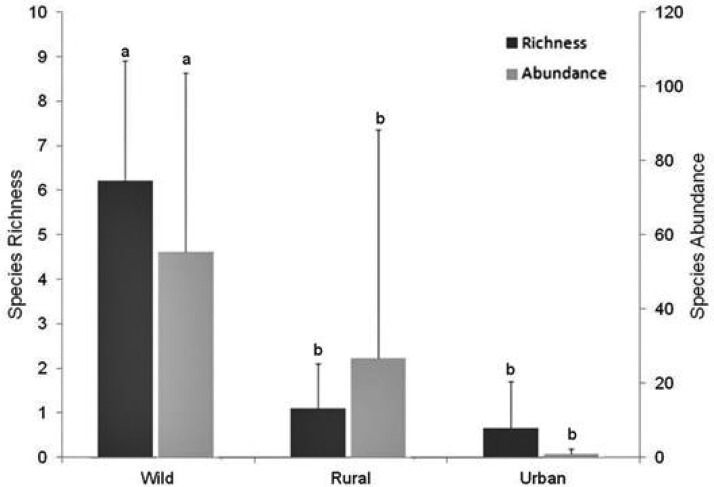
Mean species richness and abundance of individuals of phlebotomines (± SD) observed in the three environments sampled in the municipalities of the Marajó Archipelago, Pará, Brazil. Different letters over the bars indicate significantly different means, while the same letters indicate means with no significant difference.

Comparing phlebotomine fauna captured in wild, rural, and urban environments, we observed a significant difference in mean taxonomic richness (ANOVA, *F* = 24.379; df = 2, 24; *P* < 0.001). Species richness was higher in the wild environment than in rural and urban environments. No significant difference was found between the richness observed in rural and urban environments (Fig. 5).

Mean abundance of individuals also differed significantly between the environments studied (ANOVA, *F*_ _= 10.453; df = 2, 24; *P* < 0.001); the wild environment showed higher abundance of individuals, but the others didn't differ significantly (Fig. 5).

**Fig. 6:**
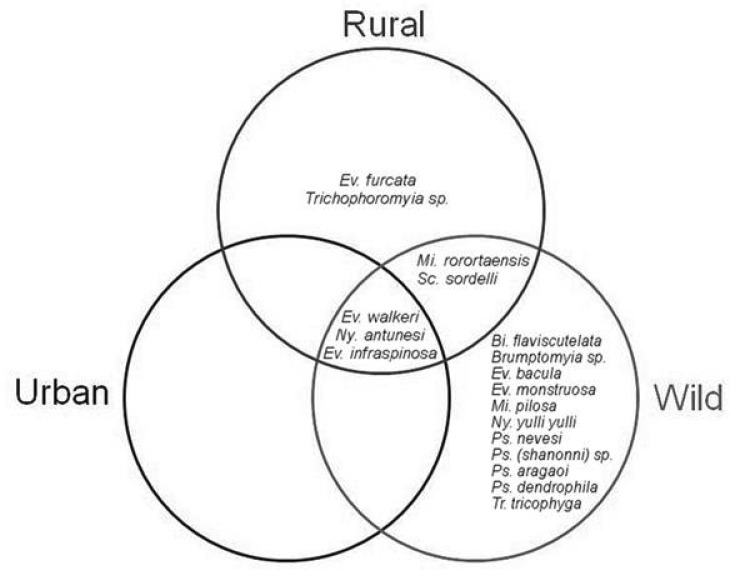
Distribution of collected species, according to sampling environments.

Phlebotomine species composition in the wild environment differed significantly from the composition observed in rural and urban environments (PERMANOVA, Pseudo-*F*_ _= 4.86; df = 2, 18; *P* = 0.001). No significant difference was observed in species composition between rural and urban environments. All species collected were found in the wild environment, except for *Ev. furcata* and *Trichophoromyia* sp., collected only in the rural environment. Two species were common between wild and rural environments, none were common to the wild and urban environments, and three occurred in the three environments (Fig. 6).

*Sciopemyia sordellii* (IndVal = 74), *Mi. rorotaensis* (IndVal = 67), *Ev. infraspinosa* (IndVal = 65), *Ny. antunesi* (IndVal = 61), and *Bi. flaviscutellata* (IndVal = 56) were strongly related to wild environment, and they were significantly singled out as indicator species of this type of environment. Analyses did not find any indicator species for rural and urban environments.

### Phlebotomines in Peri and Intradomiciliary Areas

**Fig. 7:**
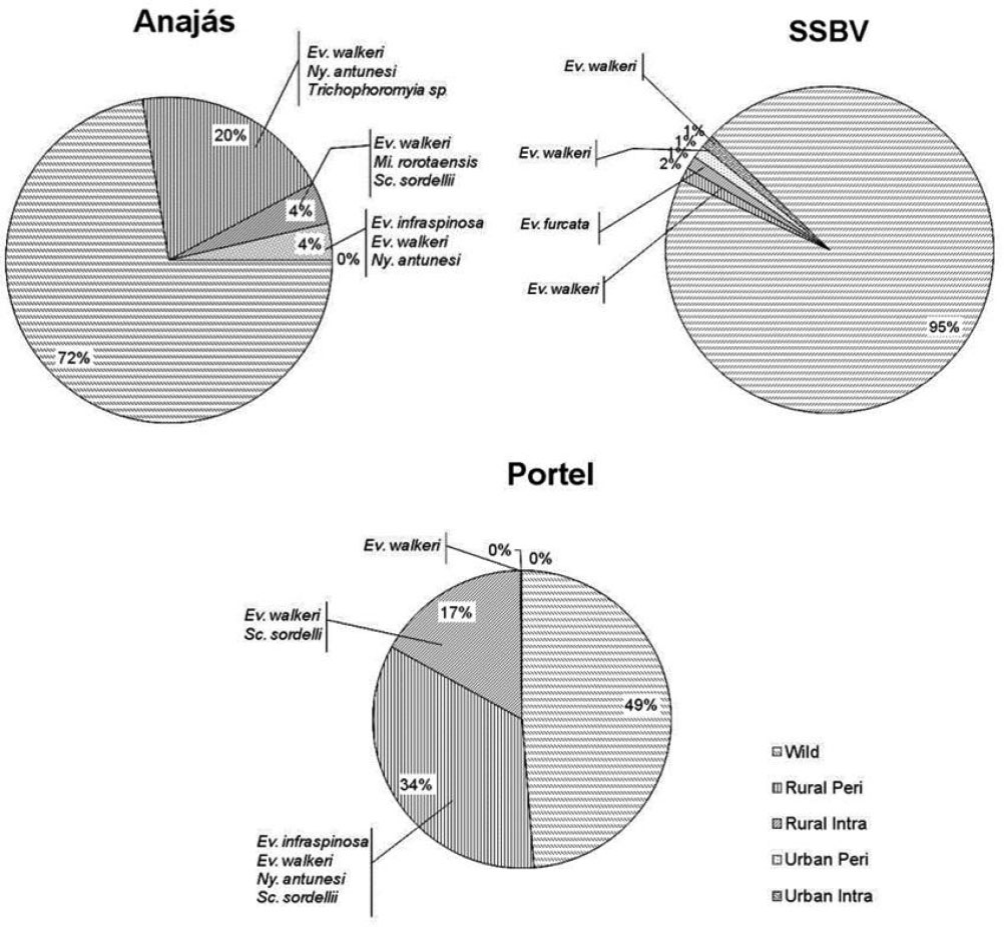
Distribution of percentage of phlebotomines captured per environment in the municipalities studied, highlighting species present in anthropized environments.

In the three municipalities studied, some phlebotomine species were observed in the rural and urban environments, both in the peri and intradomiciliary areas. São Sebastião da Boa Vista was the municipality with the smallest percentage of phlebotomines collected in anthropized environments (5%). In Anajás, we observed an expressive increase in this percentage (28%), while in Portel, more than half of phlebotomines (51%) were captured in environments which suffer great anthropic pressures (Fig. 7).

Analyzing the presence of phlebotomines inside the residences, Portel had the highest number of individuals captured in the intradomiciliary area (17%), followed by Anajás (4%) and SSBV (2%).

*Evandromyia walkeri* was the most frequent species outside the wild environment; it was present in the rural and urban environments of all municipalities, both in peri and intradomiciliary areas. The presence of *Ny. antunesi* in anthropized areas of Anajás and Portel is worth noting, due to its epidemiological importance.

## Discussion

This study provided information regarding composition and distribution of phlebotomine species in municipalities of the Marajó Island, one of the poorest areas of the Brazilian Amazon. The Marajó Archipelago is quite extensive, with relatively homogeneous phytophisionomic features and a large amount of rivers and flooded areas (PDTSAM 2007). The hydrographic characteristics of the region hinder the access to some areas, which sometimes is only possible by boat, and this explains the small amount of information on the fauna and flora of this Amazonian region. The lack of information on phlebotomine in the Marajó Archipelago increases the relevance of the data in this study from the faunistic and epidemiological standpoint.

We showed through our data that the phlebotomine fauna in the studied areas present high diversity. Species richness was high in the three municipalities, with some species predominating over others. The great majority of species identified (66.7%) had less than five individuals captured and 27.7% of the species had only one individual captured. Other studies showed a similar pattern; the phlebotomine fauna is composed of a few dominant species and a large number of species with few individuals (Pessoa et al. 2007, Silva et al. 2010).

In two of the three studied municipalities, the number of males captured was higher than females; this corroborates the results obtained in studies involving sampling of phlebotomines using CDC light trap (Dye et al. 1991, Barata et al. 2004). This result has been attributed to the phlebotomine behavior of forming male aggregations aiming to ensure mating with females (Pessoa et al. 2007).

The comparison of phlebotomine assemblages between municipalities showed that the populations were quite similar in richness, abundance, and composition. This high similarity suggests that the fauna present in the region is relatively homogeneous.

Richness and abundance were significantly higher in the wild environment than in rural and urban environments, indicating that phlebotomines species are mainly wild. Similar results have been found in studies where most phlebotomines were more frequent in forest environments than in extra-forest environments (Silva et al. 2008, Barbosa et al. 2008). This pattern was evident in the municipalities of Anajás and, mainly, São Sebastião da Boa Vista, where nearly the total (94%) specimens collected derived from the wild area. These results suggest that the specimens in these municipalities still find food and shelter in native forests (Virgens et al. 2008).

On the other hand, the number of phlebotomines collected in the rural environment in Portel was higher than in the wild environment. This municipality also showed the highest number of individuals collected. It is worth noting that, of the three municipalities sampled, Portel is the largest, most populated and has the largest urban area which leads us to infer that it also has the highest level of anthropic pressure, and consequently, of environmental changes. Several studies relate environmental changes to the urbanization process of phlebotomines (Rangel and Lainson 2009, Andrade et al. 2014) then, this fact can be influencing the high density of sandfly observed in the rural environment of Portel.

The level of integrity of native vegetation is a factor that might influence the distribution of phlebotomine species. Given the originally wild behavior of phlebotomines, their richness and abundance are directly affected by areas with preserved forests (Teodoro et al. 1993, Silva et al. 2008). Invasion of peridomiciliary areas by vectors is common in areas where primary forests have been destroyed (Lonardoni et al. 2006). In this study, phlebotomine richness and abundance in wild environments were higher than in anthropized environments (rural and urban), which can be related to the preservation level of native forests.

In all municipalities analyzed, the percentage of vegetation cover of the rural environment did not differ significantly from the wild (Fig. 2). However, despite the urban environment present smaller percentage of vegetation cover, richness, abundance, and species composition of sand flies did not differ from the rural environment (Figs. 5and 6). This similarity in the sandfly fauna between rural and urban environments may be evidencing adaptation of species to anthropogenic environments. However, it should be noted that other factors may favor the dispersion of sandflies to urban areas such as breeding in backyards of homes, which is very common in the region, even in urban areas.

The analysis of taxonomic composition showed that some species are migrating from the wild environment to other environments. *Evandromyiainfraspinosa*, *Ev. walkeri*, and *Ny. antunesi* were common to the three environments, while *Mi. rorotaensis* and *Sc. sordellii* were common to wild and rural environments. Only two species weren't collected in the wild environment (*Ev. furcata* and *Trichophoromyia* sp.), probably because they are rare, and larger samplings are required for their detection.

*Evandromyia walkeri* was the predominant species in the study area. It was present also in anthropized environments (rural and urban), including the intradomiciliary area. The great adaptability of this species was also observed by Figueira et al. (2013) in the municipality of Lábrea, Amazonas, where *Ev. walkeri* was the second most abundant species in a floodland area (várzea), mainly in the peridomiciliary area, and the main species found in an agro-forestry environment, in the municipality of Parnamirim, Rio Grande do Norte (Pinheiro et al. 2013). It is considered an opportunistic species, with eclectic feeding behavior, adjusting its habits to environmental changes and to resource availability in anthropic environments (Muniz et al. 2006). *Evandomyia walkeri* hasn't been associated to the transmission of *Leishmania* spp. to humans or other animals so far.

Phlebotomines were observed in anthropized environments (rural and urban) in the three municipalities studied and as samplings became further from the wild environment, the total number of phlebotomines decreased, mainly inside residences. Similar data were found by Feitosa and Castellon (2009) and Alves (2011).

The mean distance observed in Marajo study sites between the wild and rural environment was 494.1 m (ranging from 200.8 m to 1047.0 m) and between the wild and urban environment was 1520.6 m (ranging from 330 m to 3955 m). Studies of release and re-capture have shown that displacement capacity of sandflies varies greatly between species, ranging from sedentary species to some able to move 320 m in a single day (Alexander and Young 1992). Therefore, as this study did not seek breeding sites or immature forms within each environment, we cannot state that the sandflies in rural and urban environments do not come from wild environment. But, the high number of specimens found in the rural environment (40.7%), where there is already a strong anthropic activity, not to mention the urban environment (1.15%), suggests that sand flies are under an adaptation process, managing to survive in altered environments, using the shelters and sources of blood supply present in these areas.

The adaptive process of some phlebotomine species to new environments occurs mostly due to the devastation of large wild areas, which brings these insects to the outskirts of urban centers, making them migrate to human peri and intradomiciliary areas in search for food. Another factor that might enable the presence of phlebotomines in rural and urban environments is the large presence of chicken coops in the backyards of residences. Chicken coops in the peridomiciliary areas play an important role in the domiciliation process of species, as it is considered an attraction for these insects (Feitosa and Castellon 2009). The presence of phlebotomines in anthropized environments might be attributed to anthropic activities, associated to precarious environmental sanitation and low socioeconomic level (Martins et al. 2004, Missawa et al. 2008). All these factors are present in the studied municipalities.

Two of the species collected during this study showed epidemiological importance in leishmaniasis transmission, *Bi. flaviscutellata* and *Ny. antunesi*. *Bichromomyia flaviscutellata*, sixth overall most abundant species and second most abundant in SSBV, was captured only in the wild environment, which suggests low anthropophilia and low adaptability to altered environments. This is corroborated by other studies that describe this species as strictly wild (Marinho et al. 2008, Oliveira et al. 2011). On the other hand, this specie has been recorded in impacted areas in Brazil. Vilela et al. (2011) shows the presence of *Bi. flaviscutellata* in periurban area of a city in the North region of Brazil that was directly impacted by a hydroelectric construction, suggesting an adaptation of this species to the anthropogenic environment alterations. Besides that, Ribeiro et al. (2007) reported its presence in urban areas. This fact, associated to the high abundance of this species in the region observed in our study, has great epidemiological importance since *Bi. flaviscutellata* has been pointed out as the vector species of *Leishmania* (*Leishmania*) *amazonensis*, and an etiological agent of NWCL and diffuse cutaneous leishmaniasis (Marcondes 2001).

*Nyssomyia antunesi* was the third most abundant species, present in the wild environment, and in rural and urban environments. This species has been considered an NWCL vector, and is involved in the transmission of *Leishmania* (*Viannia*) *lindenbergi* (Lainson 2010). However, a study carried out by Ryan et al. (1984) also in the Marajó Archipelago, found three specimens of *Ny. antunesi* infected by promastigote forms of *Leishmania* (*Leishmania*) *infantum*, indicating that this species plays a potential role as VL vector. This fact is of most importance because some studies in the Brazilian Amazon region observed an urbanization trend in *Ny. antunesi* (Figueira et al. 2013). Currently, the transmission cycle of *Leishamnia infantum* in the Marajó Archipelago is considered essentially wild, involving the fox *Cerdocyon thous* and *Lutzomyia longipalpis* (Lainson et al. 1987).

The presence of NWCL vector species in the sampled areas is in accordance with the leishmaniasis occurrence pattern in the Marajó Archipelago, where the NWCL form predominates over VL. Between 2007 and 2013, 37 NWCL cases and two VL cases were reported in the municipality of Anajás, 647 NWCL cases in the municipality of Portel, and 24 NWCL cases in the municipality of São Sebastião da Boa Vista (Ministry of Health 2014). It is worth noting that the number of reported Leishmaniasis cases in the municipalities is underestimated due to the difficult access to public health services. This underreporting is particularly high regarding NWCL cases. Due to its lower lethality, NWCL is neglected by the population, who doesn’t report it to health agencies, and instead treats it with homemade medications.

The NWCL and VL cases in Brazil have been associated to agriculture, mining, and forest exploitation practices for several years (Ximenes et al. 2007). However, more and more cases have been reported in urban and periurban areas, thus evidencing a shift in the disease distribution profile (Silva-Nunes et al. 2008, Casaril et al. 2014). The presence of phlebotomines in urban areas of the three municipalities of the Marajó Archipelago evidenced the adaptation process of this vector in these areas. Further studies assessing the infection rate of these insects and the presence of infected domestic animals working as reservoirs are required to check the possibility of establishing urban transmission cycles.

The study of the phlebotomine fauna and distribution of vector (or potentially vector) species in the Marajó Archipelago is of great epidemiological relevance because it provides valuable information that might subsidize actions to control these insects in the region and, consequently, the diseases they transmit. The mapping of the distribution of species potential vector of pathogens allows the health surveillance agency direct their actions control avoiding the waste of material and human resources. Precarious socioeconomic conditions, together with intense deforestation, might be triggering the adaptation process of phlebotomine species, many of which are potential leishmaniasis vectors, to anthropized environment in Marajó Island. This emphasizes the strong relationship between the occupation of natural landscapes and disease spreading, as well as the need for monitoring these processes to maintain the health of human populations.
