# Erratum

**DOI:** 10.1093/jme/tjx113

**Published:** 2017-05-26

Correction of “Rosário, I. N. G., A. J. de Andrade, R. Ligeiro, R. Ishak, I. M. Silva. 2016. Evaluating the adaptation process of Sandfly fauna to anthropized environments in a leishmaniasis transmission area in the Brazilian Amazon. J. Med. Entomol. 54(2): 450–459.”

DOI: 10.1093/jme/tjw182

This article originally published in an unfinalized form. The article can be read in its final form online.

The publisher regrets this error.

